# Advances and challenges in biomarker guided management of triple-negative breast cancer in the era of neoadjuvant chemo-immunotherapy

**DOI:** 10.3389/fonc.2026.1842762

**Published:** 2026-07-22

**Authors:** Mariarosaria D’Amato, Maria Morritti, Franco Morelli, Luigi Ciuffreda, Marina Castelvetere, Federica Pia Mangiacotti, Barbara Pasculli, Paola Parrella

**Affiliations:** 1 Medical Oncology Unit, Fondazione Istituto di Ricovero e Cura a Carattere Scientifico (IRCCS) Casa Sollievo Della Sofferenza, San Giovanni Rotondo, FG, Italy; 2 Breast Unit, Fondazione Istituto di Ricovero e Cura a Carattere Scientifico (IRCCS) Casa Sollievo Della Sofferenza, San Giovanni Rotondo, FG, Italy; 3 Anatomo Pathology Unit, Fondazione Istituto di Ricovero e Cura a Carattere Scientifico (IRCCS) Casa Sollievo Della Sofferenza, San Giovanni Rotondo, FG, Italy; 4 Translational Medicine Department, Breast and Ovarian Cancer Research Unit, Fondazione Istituto di Ricovero e Cura a Carattere Scientifico (IRCCS) Casa Sollievo Della Sofferenza, San Giovanni Rotondo, FG, Italy

**Keywords:** triple negative breast cancer (TNBC), immune checkpoint inhibitors (ICIs), pathological complete response (pCR), neoadjuvant chemoimmunotherapy, circulating tumor DNA (ctDNA), tumor microenvironment (TME), residual cancer burden (RCB)

## Abstract

Triple-negative breast cancer (TNBC) accounts for approximately 15–20% of all breast cancers and is characterized by the absence of hormone receptors and *HER2* amplification. Early-stage TNBC is characterized by aggressive clinical behavior, high proliferative rates, early relapses, and a propensity for visceral metastases resulting in poor long-term outcomes despite initial chemosensitivity. The introduction of neoadjuvant chemo-immunotherapy, combining standard chemotherapy with immune checkpoint inhibitors (ICIs), has significantly improved pathological complete response (pCR) rates and has reshaped treatment paradigms in early TNBC. Despite these advances, a substantial proportion of patients still develop residual disease or relapse, underscoring the need for better biomarkers to guide treatment decisions. This review provides a comprehensive overview of current and emerging biomarkers in TNBC, focusing on their role in the era of neoadjuvant chemo-immunotherapy. Established clinical markers, such as pCR and residual cancer burden (RCB), remain essential for post-neoadjuvant risk stratification. Beyond these, emerging biomarkers, including tumor-infiltrating lymphocytes (TILs), programmed death ligand 1 (PD-L1), spatial immune profiling, circulating tumor DNA (ctDNA), microRNAs (miRNAs), and oncogenic signaling pathways (e.g., PI3K/AKT, MAPK, WNT/β-catenin), are under investigation for their potential to refine patient selection and predict response or resistance to therapy. The biological heterogeneity of TNBC, encompassing both tumor-intrinsic and immune microenvironment variations, contributes to differential treatment outcomes and complicates clinical decision-making. We discuss the challenges of integrating emerging biomarkers into routine clinical practice, including standardization, prospective validation, and development of combined predictive models. By summarizing both current and investigational biomarkers, this review aims to support clinicians in understanding how biomarker-driven strategies may optimize neoadjuvant therapy, reduce unnecessary toxicity, and ultimately improve long-term outcomes in TNBC.

## Introduction

Triple-negative breast cancer (TNBC) accounts for approximately 15–20% of all breast cancers and is defined by the absence of estrogen receptor (ER) and progesterone receptor (PR) expression, as well as by the lack of HER2 overexpression or gene amplification (1–3). Clinically, TNBC is characterized by aggressive behavior, high proliferative indices, early recurrence peaks, and a predilection for visceral and central nervous system metastases, resulting in inferior long-term outcomes compared with hormone receptor-positive breast cancer subtypes. Despite initial chemosensitivity, a substantial proportion of patients experience early disease relapse, particularly those with residual disease after neoadjuvant treatment (4–7). Significant improvements in response rates in the neoadjuvant treatment of TNBC have been observed with the addition of platinum agents to anthracycline- and taxane-based chemotherapy regimens (8, 9). Furthermore, the incorporation of immune checkpoint inhibitors (ICIs) into standard chemotherapy has significantly improved pathological complete response (pCR) rates and survival outcomes, establishing neoadjuvant chemo-immunotherapy as a new standard of care for patients with stage II–III TNBC (10, 11). However, these advances have introduced new clinical challenges, including treatment-related toxicity, potential overtreatment of biologically favorable tumors, and uncertainty regarding optimal post-neoadjuvant management.

In patients with residual disease after neoadjuvant therapy, adjuvant treatment decisions remain complex despite evidence supporting the use of capecitabine (12) and, in selected patients with germline *BRCA1/2* mutations, olaparib (13, 14). Notably, no prospective data are currently available to inform the optimal integration or sequencing of these agents with adjuvant immunotherapy (15–17). Furthermore, given the design of the KEYNOTE-522 trial, the benefit of adjuvant pembrolizumab in patients achieving pCR remains uncertain (11, 18, 19).

In this evolving landscape, biomarker-guided strategies are increasingly needed to refine patient selection, guide treatment escalation or de-escalation, and improve long-term outcomes while minimizing unnecessary toxicity (20). This review summarizes current evidence on biological determinants of prognosis and treatment response in early TNBC, distinguishing between biomarkers already integrated into routine clinical practice and those that remain investigational. Established tools such as pCR and residual cancer burden (RCB) are discussed alongside emerging translational biomarkers, particularly circulating tumor DNA (ctDNA) and tumor-infiltrating lymphocytes (TILs), which may improve post-neoadjuvant risk stratification and guide personalized treatment strategies. Particular attention is also given to early translational biomarkers, including microRNAs (miRNAs) and spatial transcriptomics, which may represent future directions in TNBC precision medicine.

**Figure 1:**
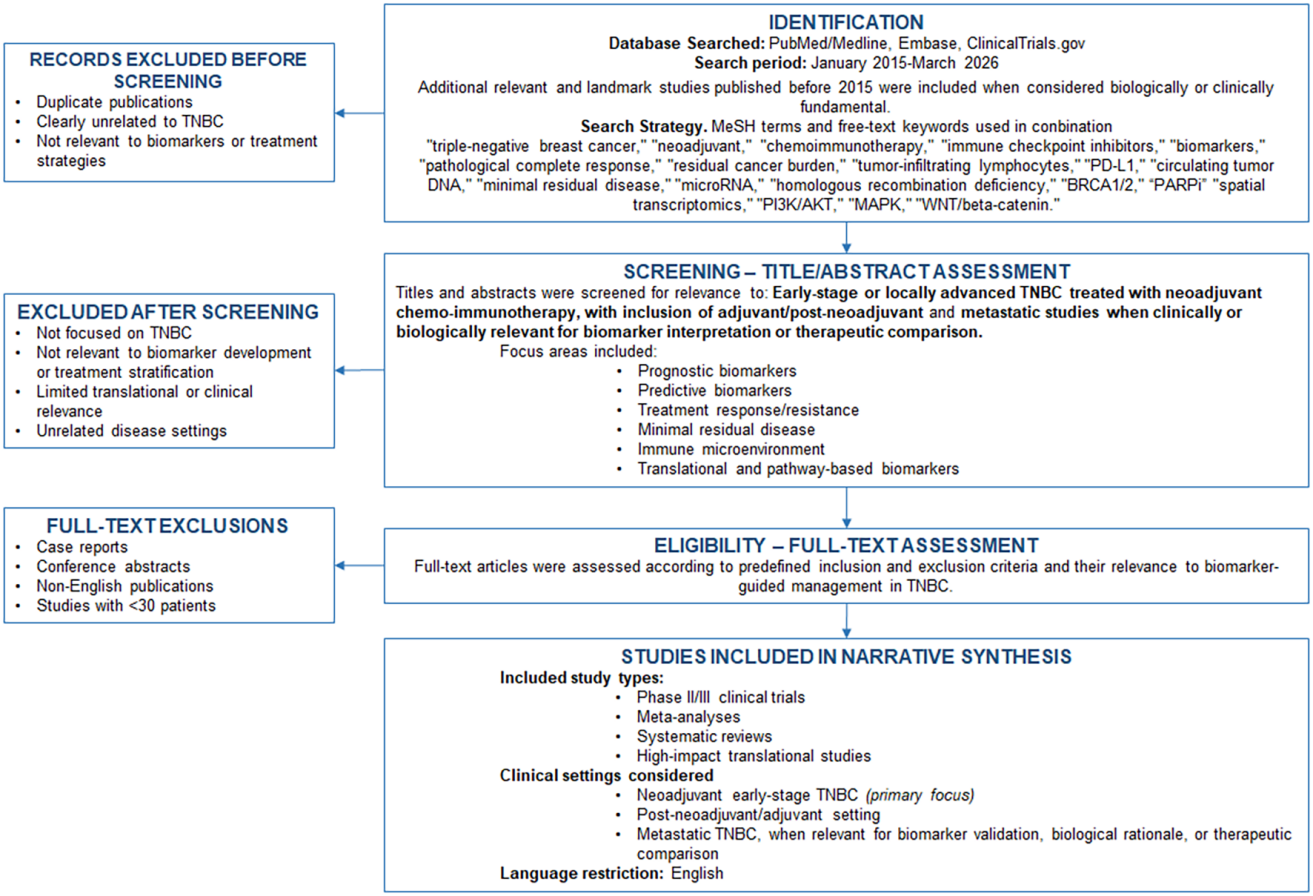
Study selection and literature search strategy for biomarkers in triple-negative breast cancer. Flow diagram illustrating the systematic identification, screening, eligibility assessment, and inclusion of studies. Literature was searched in PubMed/Medline, Embase, and ClinicalTrials.gov for the period January 2015–March 2026, using predefined MeSH terms and free-text keywords related to TNBC, immunotherapy, chemotherapy response, tumor microenvironment, and molecular biomarkers.

This review was conducted as a narrative synthesis with predefined systematic elements. A structured literature search was performed in PubMed/MEDLINE, Embase, and ClinicalTrials.gov, as detailed in **Figure 1**. Eligible studies included phase II and III clinical trials, meta-analyses, systematic reviews, and high-impact translational studies focusing on TNBC and published in English. Case reports, conference abstracts, and studies enrolling fewer than 30 patients were excluded to ensure methodological rigor and clinical relevance.

## Biological landscape of TNBC

Large-scale genomic and transcriptomic profiling studies have consistently demonstrated that TNBC is not a single disease entity, but rather a biologically heterogeneous spectrum of tumors characterized by distinct oncogenic programs and complex interactions with the tumor microenvironment (TME) (21–26). A central hallmark of TNBC biology is genomic instability, which underpins its molecular heterogeneity and contributes to its aggressive clinical behavior (27). Alterations in DNA damage response (DDR) pathways are highly prevalent in TNBC, particularly those affecting homologous recombination repair (HRR) (21, 28, 29). While *BRCA1/2* mutations play a pivotal role, HRR defects may also arise through epigenetic silencing or alterations in other HRR-related genes, resulting in a broader homologous recombination deficiency (HRD) phenotype (30, 31). In the context of these underlying molecular alterations, HRD is associated with genomic scarring and may contribute to activation of innate immune sensing pathways, such as cGAS–STING (32, 33). This, in turn, contributes to increased tumor immunogenicity and may enhance responsiveness to chemo-immunotherapy as well as sensitivity to DNA-damaging agents and PARP inhibitors (PARPi) (27, 30, 31).

From an immunological perspective, TNBC also exhibits marked heterogeneity in the composition of the tumor immune microenvironment (TME). Tumors may display inflamed, immune-desert, or immune-excluded phenotypes, characterized by active cytotoxic immune infiltration, minimal immune presence, or stromal-restricted immune cells, respectively, with corresponding differences in prognosis and therapeutic sensitivity (11, 34, 35). Importantly, these immune states are dynamic and may be modulated by systemic therapies, including chemotherapy-induced immunogenic cell death, providing a biological rationale for chemo-immunotherapy combinations (35, 36).

Building on the biological complexity of TNBC, transcriptomic profiling has enabled the identification of molecular subgroups that further capture tumor heterogeneity and may provide biologically and potentially clinically relevant insights (**Table 1**). Among the earliest and most widely adopted frameworks, the classification proposed by Lehmann et al. (22) identified six transcriptomic subtypes: basal-like 1 (BL1), basal-like 2 (BL2), mesenchymal (M), mesenchymal stem-like (MSL), luminal androgen receptor (LAR), and immunomodulatory (IM). Subsequent refinements and integrative analyses have further emphasized immune-related distinctions, including basal-like immune-activated (BLIA) and basal-like immune-suppressed (BLIS) tumors, although the clinical reproducibility and standardization of these classifications remain under investigation (22, 23, 25, 37).

**Table 1:** Molecular subtypes and biologically relevant TNBC subgroups with associated pathways.

Subgroup	Key features	TME	Clinical relevance
Basal-like Immune-Activated (BLIA)	Immune gene enriched signature; HRD/BRCA subset.	Inflamed; high CD8^+^ T cell infiltrates; PD-L1 expression.	Higher pCR rates; potential sensitivity to ICIs.
Basal-like Immune-Suppressed (BLIS)	Low immune gene expression; proliferative programs	Immune-desert or excluded; low TILs	Lower pCR; limited ICIs benefit
Mesenchymal (M)	EMT activation; PI3K/AKT pathway enrichment	Often immune-excluded	Intermediate chemotherapy response
Luminal Androgen Receptor (LAR)	AR signaling; low proliferation	Immune-cold	Lower pCR; AR-targeted therapies investigational
HRD/BRCA-mutated TNBC *(biologically relevant subgroup)*	DNA repair deficiency; genomic instability	Often inflamed; variable TILs	Sensitivity to platinum and PARP inhibitors
PD-L1–High TNBC *(biomarker defined subgroup)*	Cross-subtype biomarker	Increased PD-L1 on tumor/immune cells	Predictive of ICIs benefit in metastatic TNBC, not validated in early TNBC.

AKT (PKB) protein kinase B; AR, androgen receptor; BRCA1/2, breast cancer susceptibility gene 1/2; EMT, epithelial–mesenchymal transition; HRD, homologous recombination deficiency; ICIs, immune checkpoint inhibitors; PARP, poly(ADP-ribose) polymerase; pCR, pathological complete response; PD-L1, programmed death-ligand 1; PI3K, phosphoinositide 3-kinase; TILs, tumor-infiltrating lymphocytes; TME, tumor microenvironment; TNBC, triple-negative breast cancer.

BLIA tumors are typically associated with high proliferative activity, frequent homologous recombination deficiency (HRD) or *BRCA1/2* alterations, and, in some cases, increased tumor mutational burden (TMB), alongside an immune-inflamed microenvironment enriched in CD8^+^ tumor-infiltrating lymphocytes (TILs) and PD-L1 expression (23, 38). These characteristics have been associated with higher pCR rates following neoadjuvant chemotherapy and more favorable survival outcomes, particularly in immune-enriched TNBC characterized by elevated TILs levels and immune activation (23, 38). Emerging evidence further suggests that these tumors may derive greater benefit from immune checkpoint inhibitors (11). By contrast, BLIS tumors exhibit reduced immune-related gene expression, enrichment of proliferation- and cell cycle-related pathways, limited immune infiltration, and poorer clinical outcomes (23, 24).

The luminal androgen receptor (LAR) subtype represents a biologically distinct entity driven by androgen receptor (AR) signaling, with luminal-like transcriptional features, lower proliferative activity, and a relatively immune-cold microenvironment characterized by limited inflammatory signaling (22–24). Clinically, LAR tumors have generally been associated with lower pCR rates after neoadjuvant chemotherapy, while AR-targeted therapeutic strategies remain under active investigation (39, 40). Mesenchymal (M) tumors are enriched for epithelial–mesenchymal transition (EMT), stemness-associated programs, and PI3K/AKT/mTOR signaling, although their clinical implications and therapeutic vulnerabilities remain less well defined (22, 23).

Beyond transcriptomic classifications, biologically relevant subgroups defined by genomic and immune biomarkers, including HRD/*BRCA-*mutated and PD-L1–high tumors, may further refine therapeutic stratification. HRD-positive and *BRCA1/2*-mutated TNBCs have been associated with increased sensitivity to platinum-based chemotherapy and PARP inhibitors (13, 14, 41, 42). PD-L1 expression may also identify subsets more likely to benefit from immune checkpoint inhibitors, although its predictive role in early-stage TNBC remains incompletely defined (11, 35, 43).

Current transcriptomic classifications have not yet been fully standardized across platforms and may be influenced by intratumoral heterogeneity and treatment-induced plasticity, limiting their immediate applicability in routine clinical practice. In addition, these frameworks only partially capture the complexity of TNBC biology, as they do not fully incorporate spatial immune organization or dynamic biomarkers of treatment response. Consequently, increasing attention has shifted toward integrated biomarker approaches that combine tumor-intrinsic features with pathological and immune-related parameters to refine risk stratification and therapeutic decision-making in early TNBC, particularly in the neoadjuvant and post-neoadjuvant settings.

## Evolution of neoadjuvant therapy in TNBC

For several decades, anthracycline- and taxane-based chemotherapy has constituted the backbone of neoadjuvant therapy for early-stage TNBC. The addition of platinum agents, particularly carboplatin, has consistently demonstrated an ability to enhance pCR. A meta-analysis of neoadjuvant platinum-based trials reported an increase in pCR rates from 37.0% to 52.1% (OR 1.96; 95% CI 1.46–2.62; p<0.001), although this did not translate into a statistically significant improvement in event-free survival (EFS) (HR 0.72; 95% CI 0.49–1.06; p=0.094) (8). Randomized trials have further refined the role of platinum compounds in this setting. In patients with TNBC enrolled in GeparSixto, the addition of carboplatin to anthracycline/taxane-based neoadjuvant chemotherapy resulted in significant improvements in disease-free survival (DFS) (HR 0.56; 95% CI 0.34–0.93; p=0.022) and distant DFS (HR 0.50; 95% CI 0.29–0.86; p=0.013) at a median follow-up of 47.3 months (44). Similarly, CALGB 40603 (41) and BrighTNess (42) demonstrated substantial increases in pCR rates with carboplatin (54% vs 41% and 58% vs 31%, respectively), although long-term outcome benefits remain less consistent. Notably, the BrighTNess trial showed no additional benefit from the addition of the PARP inhibitor veliparib beyond carboplatin-containing chemotherapy (42).

The phase II I-SPY2 trial provided initial proof-of-concept for immunotherapy, demonstrating a marked increase in estimated pathologic complete response (pCR) rates with the addition of pembrolizumab to standard neoadjuvant chemotherapy (approximately 60% vs 22%) (10). This benefit was subsequently confirmed in the phase III KEYNOTE-522 trial, in which pembrolizumab combined with neoadjuvant chemotherapy (carboplatin/paclitaxel followed by anthracycline/cyclophosphamide) significantly improved pCR (64.8% vs 51.2%) and EFS (HR 0.63; 95% CI 0.48–0.82; p<0.001) in stage II–III TNBC (18). Updated analyses demonstrated a sustained overall survival (OS) benefit (HR 0.66; 95% CI 0.50–0.87; p=0.0015), establishing pembrolizumab as the standard immunotherapeutic backbone in the neoadjuvant setting regardless of PD-L1 status or clinical subgroups (19). Comparable findings were observed with other checkpoint inhibitors. In IMpassion031, the addition of atezolizumab significantly increased pCR rates (57.6% vs 41.1%; p=0.0044) (45) while GeparNuevo, evaluating durvalumab, did not meet its primary pCR endpoint but showed favorable long-term improvements in invasive disease-free survival (DFS), distant DFS, and OS (46). This variability is further supported by early-phase translational evidence, including the NeoPACT trial, which reported promising activity of neoadjuvant pembrolizumab combined with carboplatin and docetaxel, while also highlighting biological heterogeneity in treatment response across molecularly and immune-defined subgroups (47).

The post-neoadjuvant setting is primarily defined by risk stratification after surgery, particularly in patients with residual disease or other high-risk features. In KEYNOTE-522, pembrolizumab was continued postoperatively, supporting the concept of perioperative immunotherapy (18). However, the extent to which adjuvant pembrolizumab contributes to clinical benefit, particularly among patients achieving pCR, remains uncertain and is currently being explored in treatment de-escalation strategies. This question is particularly relevant given the non-negligible incidence of immune-related adverse events, reported in approximately 33.5% of patients receiving pembrolizumab compared with 11.3% in the chemotherapy-alone group (18). The phase III CREATE-X trial demonstrated that adjuvant capecitabine significantly improved disease-free survival (HR 0.58; 95% CI 0.39–0.87) and OS (HR 0.52; 95% CI 0.30–0.90), with the greatest benefit observed in the TNBC subgroup (12). Similarly, the OlympiA trial established adjuvant olaparib as a standard treatment option for patients with germline *BRCA1/2* mutations and high-risk *HER2*-negative disease, including TNBC (13). However, the optimal sequencing and integration of capecitabine, PARP inhibitors, and immunotherapy after neoadjuvant checkpoint blockade remain undefined, further emphasizing the need for biomarker-driven strategies to guide post-neoadjuvant treatment selection.

## Clinically implemented biomarkers

**Figure 2:**
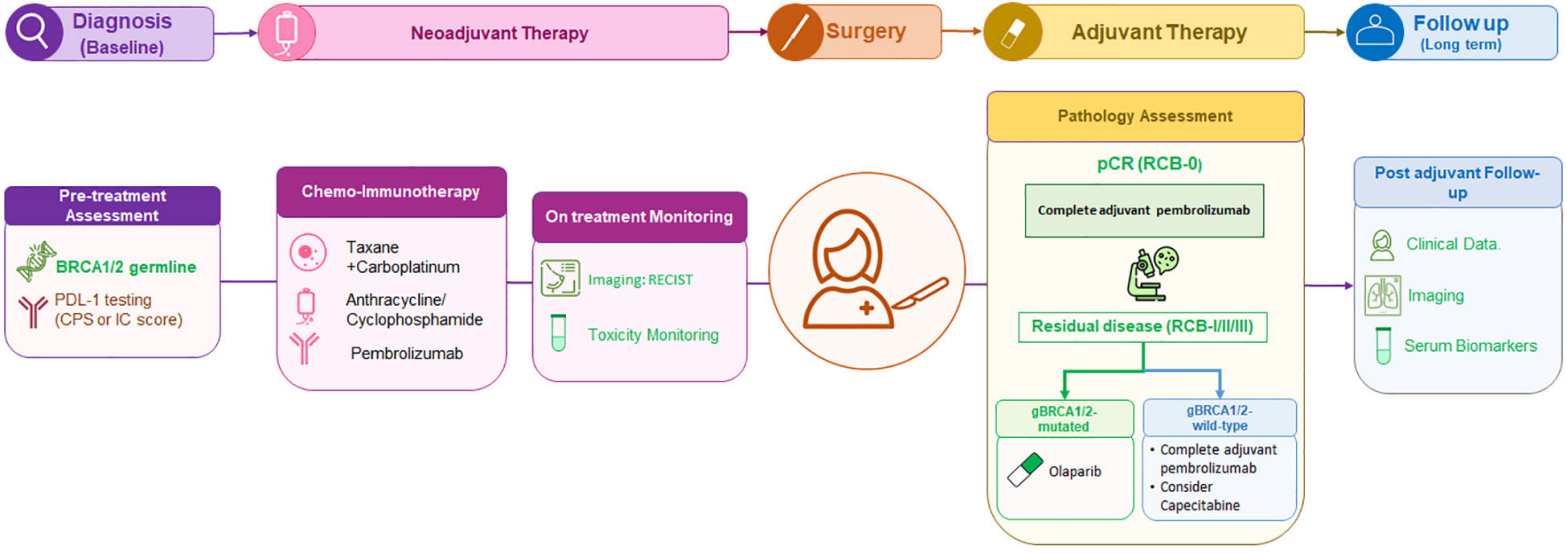
Current biomarker-informed treatment framework in early triple-negative breast cancer (TNBC). Schematic overview of the current clinical management pathway for early TNBC, from diagnosis through neoadjuvant therapy, surgery, adjuvant treatment, and follow-up. The figure highlights biomarkers currently incorporated into clinical decision-making, including germline BRCA1/2 testing and pathological response assessment. Post-neoadjuvant treatment adaptation is guided by pCR/RCB status and germline BRCA1/2 status, supporting the use of therapies such as pembrolizumab, capecitabine, or olaparib according to recurrence risk and residual disease burden.

The growing recognition of biological heterogeneity in early TNBC has underscored the need for robust biomarkers to improve risk stratification and guide therapeutic decision-making across the neoadjuvant and post-neoadjuvant settings (**Figure 2**). Clinically implemented biomarkers include pathological response measures, DNA damage response alterations, and PD-L1 expression, each with distinct roles in prognosis, risk stratification, and therapeutic guidance (**Table 2**). Together, these biomarkers constitute the current foundation of biomarker-guided management in early-stage TNBC.

**Table 2:** Clinically implemented biomarkers in early stage TNBC.

Biomarker	Clinical role	Guideline status	Clinical implication
BRCA1/2 *(germline)*	Selection for targeted therapy	Standard of care.	Guides use of PARP inhibitors (olaparib) in eligible patients
pCR	Surrogate endpoint of treatment response after NACT	Standard of care	Prognostic for EFS/OS; individual-level prediction limited
RCB	Quantification of residual disease after NACT	Clinically Recommended.	Risk stratification for relapse based on residual burden
PD-L1	Predictive biomarker for immune checkpoint inhibitors	Not required for treatment selection in early TNBC	Used mainly in metastatic setting; role in early TNBC remains investigational

BRCA1/2, breast cancer gene 1/2; EFS, event-free survival; NACT, neoadjuvant chemotherapy; OS, overall survival; PARP, poly(ADP-ribose) polymerase; PD-L1, programmed death-ligand 1; pCR, pathological complete response; RCB, residual cancer burden; TNBC, triple-negative breast cancer.

### Pathological response and residual cancer burden

pCR, defined as the absence of residual invasive disease in both the breast and axillary lymph nodes at surgery (ypT0/is ypN0), remains one of the most widely used surrogate endpoints in the neoadjuvant treatment setting. Across breast cancer subtypes, achievement of pCR has consistently been associated with favorable long-term outcomes. In the pooled analysis by Cortazar et al. (7), including 11,955 patients from 12 international neoadjuvant trials, pCR was significantly associated with improved EFS and OS, with the strongest prognostic associations observed in biologically aggressive subtypes, particularly TNBC and HER2-positive disease. Importantly, although pCR was prognostic at the individual-patient level, the analysis did not support pCR as a validated surrogate endpoint for long-term outcomes at the trial level, underscoring the complexity of interpreting pCR in drug development. Notably, the prognostic association between pCR and survival was substantially stronger in TNBC than in hormone receptor–positive/HER2-negative disease, underscoring the biological relevance of treatment response in this subtype.

Despite its clinical utility, pCR has important limitations. As a binary endpoint, it does not quantify residual disease burden or capture its biological heterogeneity, nor does it adequately capture occult systemic disease that may persist following surgery. To address these limitations, the RCB index was developed as a standardized quantitative framework for pathological assessment following neoadjuvant therapy (48–50). By integrating residual tumor dimensions, tumor cellularity, and nodal involvement into a continuous score, RCB stratifies patients into four prognostic categories ranging from RCB-0 (equivalent to pCR) to RCB-III.

Among breast cancer subtypes, RCB has demonstrated greater prognostic resolution than binary response measures such as pCR alone. In a large pooled multicenter analysis of 5,161 patients treated with neoadjuvant chemotherapy, increasing RCB category showed a strong and continuous association with progressively worse survival outcomes across all major breast cancer subtypes (51). The prognostic value of RCB is particularly pronounced in TNBC, where increasing residual disease burden is associated with progressively worse outcomes. Five-year DRFS/RFS is highest in RCB-0/I and markedly lower in RCB-II and RCB-III (6, 52, 53).

In the chemo-immunotherapy setting, exploratory analyses from KEYNOTE-522 suggest that RCB may retain prognostic relevance among patients treated with pembrolizumab-based neoadjuvant therapy (54). In this pre-specified exploratory analysis including 1,174 patients with stage II–III TNBC, pembrolizumab was associated with a shift toward lower RCB categories, with a higher proportion of patients achieving RCB-0 and fewer patients classified as RCB-I to RCB-III compared with placebo (54). Event-free survival (EFS) remained associated with residual disease burden across RCB categories. The greatest estimated EFS benefit with pembrolizumab was observed in patients with RCB-II disease (HR 0.52, 95% CI 0.32–0.82), whereas effects appeared less pronounced and statistically uncertain in the RCB-0 (HR 0.70, 95% CI 0.38–1.31), RCB-I (HR 0.92, 95% CI 0.39–2.20), and RCB-III (HR 1.24, 95% CI 0.69–2.23) subgroups, although interpretation is limited by the exploratory nature of these analyses (54).

Consistent with these data, ESMO Clinical Practice Guidelines and NCCN Guidelines, support escalation strategies for patients with residual invasive disease after neoadjuvant therapy, particularly in TNBC, while emphasizing that post-neoadjuvant management should incorporate tumor biology and residual disease burden rather than rely exclusively on pCR (15, 16). Conversely, de-escalation strategies in patients achieving pCR or Minimal Residual Disease (MRD) remain investigational.

**Figure 3:**
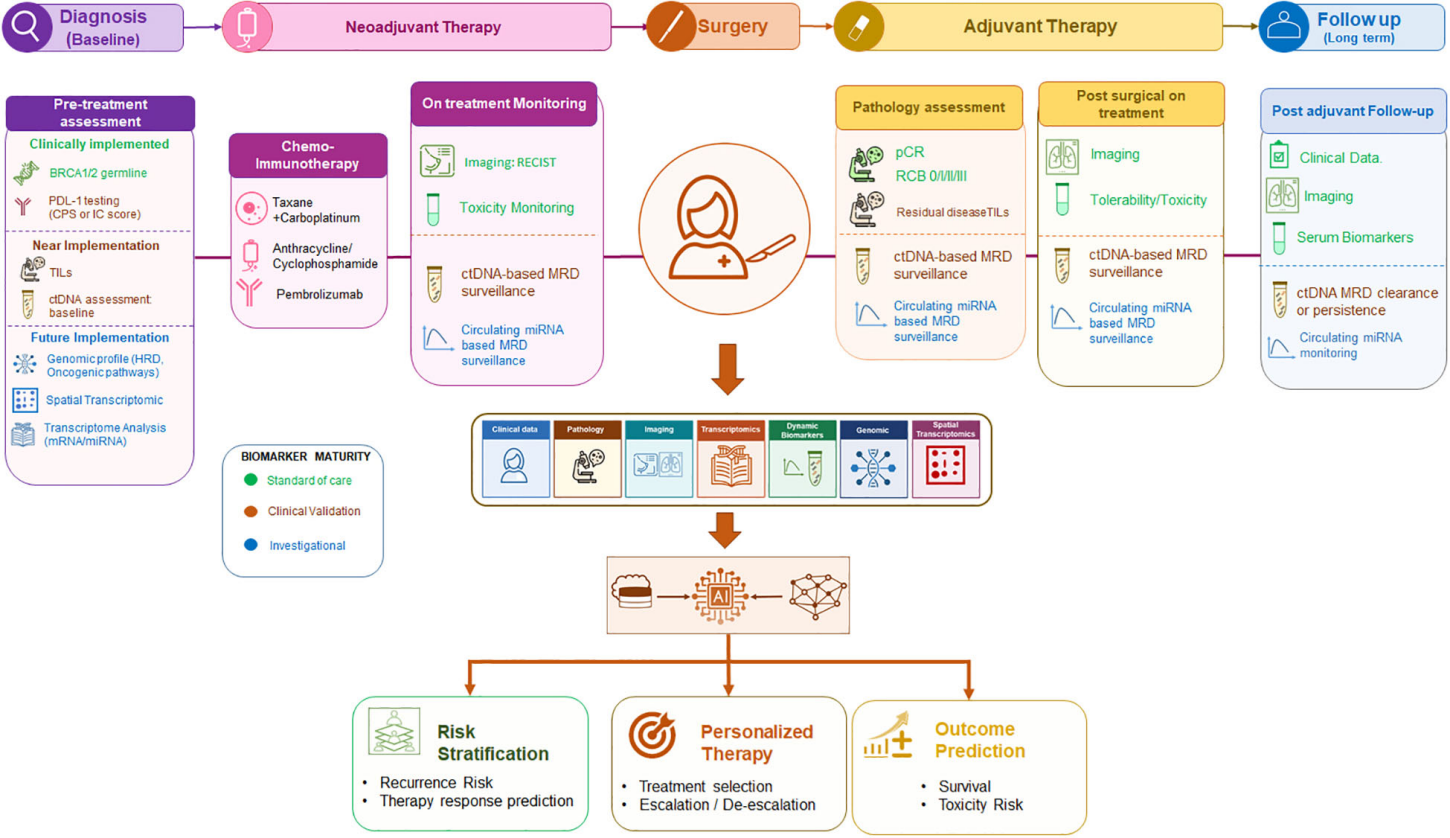
Toward a longitudinal, biomarker-informed, adaptive model of care in early TNBC. Schematic representation of an integrated framework combining clinical, pathological, immune, and molecular biomarkers across the disease continuum in early triple-negative breast cancer (TNBC). At diagnosis, established and emerging biomarkers, including TILs and baseline ctDNA, are integrated, with additional features such as genomic alterations and transcriptomic profiles anticipated to further refine risk stratification. Across all time points, multimodal data are integrated using machine learning and artificial intelligence to dynamically update individualized risk profiles. During neoadjuvant therapy, serial assessments through imaging and liquid biopsy–derived biomarkers may provide insights into treatment response, resistance, and tumor evolution. After surgery, RCB and MRD, together with additional molecular biomarkers, may improve prognostication and guide post-neoadjuvant treatment decisions. The icons used to create figures were obtained from Flaticon and are provided under its free-use license (https://www.flaticon.com/icons).

Despite its prognostic performance, RCB remains a static histopathological measure that does not fully capture subclinical systemic disease or tumor evolutionary dynamics. In particular, it cannot directly assess molecular residual disease that may persist despite low or absent histological tumor burden. These limitations have prompted increasing interest in integrating RCB with dynamic biomarkers such as ctDNA, which may provide complementary information on MRD, disease kinetics, and clonal evolution (55–57). Future risk stratification models will likely evolve toward multimodal frameworks integrating pathological, clinical, and molecular biomarkers to improve prognostic precision and guide post-neoadjuvant therapeutic decision-making in TNBC (**Figure 3**).

### DNA damage response alterations

Germline *BRCA1/2* testing is recommended in patients with early-stage TNBC according to current ESMO, NCCN, and ASCO guidelines (15–17), given its clinical implications for both hereditary cancer risk assessment and therapeutic decision-making. The clinical relevance of this pathway is supported by the established efficacy of PARP inhibition in germline *BRCA-*mutated *HER2-*negative breast cancer. In the metastatic setting, randomized trials have demonstrated improved outcomes with PARP inhibitors compared with standard chemotherapy (58), while in early-stage disease the phase III OlympiA trial showed that adjuvant olaparib significantly improved invasive disease-free survival (HR 0.58; 95% CI 0.41–0.82) (13) and overall survival (HR 0.68; 95% CI 0.50–0.91) (14) in patients with high-risk germline *BRCA1/2-*mutated breast cancer. These findings established adjuvant olaparib as a standard treatment option for high-risk germline *BRCA1/2-*mutated *HER2-*negative breast cancer, reinforcing the recommendation for routine germline testing in TNBC.

Beyond olaparib, other PARP inhibitors have been evaluated in early-stage disease, although their role remains investigational (59). Talazoparib has demonstrated efficacy in metastatic germline *BRCA-*mutated breast cancer in the phase III EMBRACA trial (progressiojn Free Survival, PFS HR 0.54; 95% CI 0.41–0.71) (60). In the early TNBC setting, phase II and exploratory neoadjuvant studies have reported pCR rates ranging from approximately 45% to 53%, suggesting antitumor activity in highly selected populations. However, these studies are predominantly single-arm and do not allow robust comparison with standard chemotherapy, limiting interpretation of long-term benefit (60–65).

Platinum-based chemotherapy is also widely used in TNBC due to its association with genomic instability and defective DNA repair mechanisms. Across neoadjuvant studies, the addition of platinum agents has been shown to increase pCR rates. However, this benefit is not restricted to germline *BRCA-*mutated tumors, suggesting that platinum sensitivity extends beyond *BRCA*- status alone and likely reflects broader determinants of genetic instability and basal-like tumor biology (41, 66, 67), To refine patient selection beyond germline *BRCA1/2* status, HRD assays have been developed as composite measures of genomic scarring (30). However, their clinical utility in early-stage breast cancer remains limited by inter-assay variability across commercial platforms, including Myriad myChoice, AmoyDx, and Foundation Medicine, each employing distinct genomic instability scoring systems and non-equivalent thresholds, as well as non-standardized cut-offs and incomplete prospective validation (29, 68, 69). Consistent with this, pCR have been observed in both HRD-positive and HRD-negative tumors across studies, further underscoring the insufficient discriminatory performance of current HRD classifiers for routine therapeutic stratification in early TNBC (67, 70–72).

### PD-L1 expression

PD-L1 expression is frequently assessed in early TNBC, although in the neoadjuvant setting it is not currently used as an independent determinant of treatment selection (15). Biologically, PD-L1 is expressed on both tumor cells and tumor-infiltrating immune cells and plays a central role in immune evasion through inhibition of T-cell activation. Its expression is largely driven by interferon-γ–mediated signaling within an inflamed tumor microenvironment, consistent with an adaptive immune resistance mechanism rather than a fixed tumor-intrinsic feature (73).

This biological rationale has translated into clinical evaluation of immune checkpoint inhibition in early TNBC. In the neoadjuvant KEYNOTE-522 trial, pembrolizumab improved pCR rates in both PD-L1–positive (CPS ≥1: 68.9% vs 54.9%) and PD-L1–negative (45.3% vs 30.3%) subgroups, suggesting that PD-L1 expression is not required for clinical benefit in this setting (11, 18). Similarly, in IMpassion031, atezolizumab increased pCR rates irrespective of PD-L1 status, both in PD-L1–positive (69% vs 49%) and PD-L1–negative patients (48% vs 34%), with longer-term follow-up showing a numerically consistent but not statistically significant trend toward improved EFS regardless of PD-L1 status (HR 0.76; 95% CI 0.47–1.21) (45, 74).

In contrast, PD-L1 retains a predictive role in metastatic TNBC. In KEYNOTE-355, pembrolizumab plus chemotherapy demonstrated clinically meaningful benefit primarily in PD-L1–positive tumors defined by CPS ≥10 (43), while IMpassion130 reported improved outcomes with atezolizumab predominantly in PD-L1–positive disease as defined by the PD-L1 immune-cell (IC) scoring system (75).

Overall, available evidence suggests that PD-L1 has limited utility as a standalone biomarker for treatment selection in early-stage TNBC, in contrast to the metastatic setting where PD-L1 enrichment appears more relevant for patient selection. This discrepancy likely reflects fundamental differences in tumor–immune dynamics across disease stages, as well as the immunomodulatory effects of concurrent chemotherapy in the neoadjuvant context (36). Additional complexity arises from inter-assay variability between CPS and IC scoring systems, the dynamic nature of PD-L1 expression, and the absence of a universally validated positivity threshold, all of which further limit its standardization as a predictive biomarker in early TNBC (76).

## Emerging immune biomarkers

The immune microenvironment of TNBC is a major determinant of response to neoadjuvant chemo-immunotherapy and has therefore emerged as a central focus of translational research (**Table 3**). Immune biomarkers encompass a spectrum of candidates with varying degrees of clinical maturity, ranging from the established prognostic role of tumor-infiltrating lymphocytes (TILs) to the more exploratory applications of spatial immune profiling and characterization of immune functional states (**Figure 3**). Together, these approaches aim to improve understanding of antitumor immunity and refine patient stratification beyond conventional clinicopathological factors.

**Table 3:** Emerging biomarkers in early stage TNBC.

Biomarker	Biological role	Development stage	Clinical relevance
TILs	Adaptive immune infiltration in TME	Clinical Validation	Prognostic; associated with pCR; no validated thresholds for treatment selection.
Transcriptomic signatures	Gene Expression profile (e.g. TNBC-DX)	Clinical validation	Prognostic; no validated in prospective cohorts
Spatial Transcriptomics	Spatial mapping of TME architecture	Investigational	Defines immune phenotypes linked to response; not clinically applicable
ctDNA	Circulating tumor-derived DNA; disease burden dynamics	Clinical validation.	MRD and relapse prediction; not yet standardized for clinical decision-making
HRD score	DNA repair deficiency/genomic instability	Clinical validation.	Predictive of platinum/PARP sensitivity; limited by assay heterogeneity
PI3K/AKT pathway alterations	Oncogenic signaling pathway activation	Investigational	Investigational in early TNBC; not clinically actionable
miRNA signatures *(tissue/circulating)*	Post-transcriptional regulation of EMT and immunity	Investigational	Promising prognostic/MRD markers; lack of standardization

ctDNA, circulating tumor DNA; EMT, epithelial-to-mesenchymal transition; HRD, homologous recombination deficiency; miRNA, microRNA; MRD, minimal residual disease; pCR, pathological complete response; PI3K, phosphoinositide 3-kinase; TME, tumor microenvironment; TILs, tumor-infiltrating lymphocytes, TNBC Triple Negative breast Cancer.

### Tumor-infiltrating lymphocytes

TILs are mononuclear immune cells, predominantly T lymphocytes, with variable contributions from B cells and natural killer cells, that accumulate within the tumor microenvironment (TME) in response to tumor-associated antigenic stimulation and chemokine-mediated recruitment (77, 78). Their presence is thought to reflect an active host–tumor immune interaction characterized by antigen recognition, endothelial transmigration, and clonal expansion of tumor-reactive lymphocytes. Histopathologically, TILs are evaluated on hematoxylin and eosin (H&E)-stained sections and stratified into stromal TILs (sTILs), located within the tumor-associated stroma, and intratumoral TILs (itTILs), residing within tumor cell nests. Of these, stromal TILs are generally considered the most reproducible and clinically validated parameter and are recommended for routine assessment according to the standardized guidelines of the International TILs Working Group (77, 79–81). Quantification is expressed as the percentage of stromal area occupied by mononuclear inflammatory cells, with tumors commonly classified as TIL-low (<10%), intermediate (10–59%), or TIL-high (≥60%), although these thresholds are primarily used for research stratification and no universally validated clinically actionable cut-offs have been established (38, 79).

Beyond quantitative assessment, the TIL compartment appears biologically heterogeneous, and its functional impact may be governed by the balance between effector and regulatory subsets (82). CD8^+^ cytotoxic T cells are generally regarded as the principal effector population mediating tumor cell lysis via perforin/granzyme release and Fas–FasL signaling, whereas CD4^+^ Th1 cells are thought to sustain antitumor immunity through interferon-γ–mediated potentiation of cytotoxic responses. Conversely, FOXP3 positive regulatory T cells may exert immunosuppressive effects, although their prognostic significance remains context-dependent and incompletely understood (78, 81, 83). Collectively, these observations suggest that qualitative immune composition, rather than absolute lymphocytic density alone, may be an important determinant of biological activity within the TME.

Clinically, high baseline stromal TIL levels have been associated with improved outcomes in TNBC, including higher rates of pCR following neoadjuvant chemotherapy and more favorable long-term survival (84, 85). In a pooled analysis of 3,771 patients, each 10% increment in stromal TILs was associated with improved disease-free survival (HR 0.93, 95% CI 0.87–0.98) and overall survival (HR 0.92, 95% CI 0.86–0.99) (38). Although statistically robust, the magnitude of these associations is modest at the individual patient level, and clinical translation remains constrained by inter-observer variability and the absence of standardized, actionable thresholds. Nonetheless, this prognostic relevance has been sufficiently consistent to warrant endorsement by major international guidelines, which recognize stromal TILs as a validated prognostic biomarker in early-stage TNBC (15–17).

The predictive value of TILs represents a distinct and less established dimension. In the randomized neoadjuvant trials KEYNOTE-522 and IMpassion031, the addition of ICIs appeared to improve pCR rates across baseline TIL strata, indicating that TILs do not currently reliably identify which patients benefit from immunotherapy over and above chemotherapy alone (11, 35, 45). Discordant clinical outcomes within TIL-defined subgroups further suggest that quantitative immune infiltration alone is insufficient to capture functional immune competence. Failure of response in TIL-rich tumors could reflect T-cell exhaustion, immunosuppressive myeloid and regulatory T-cell activity, or upregulation of inhibitory immune checkpoints. Accordingly, major guidelines explicitly distinguish between the prognostic and predictive roles of TILs, endorsing the former while cautioning against their use as predictive markers for immunotherapy selection in routine clinical practice (15–17).

Beyond their role as a static baseline parameter, TILs also appear to carry prognostic information when assessed in the post-treatment setting. *Post hoc* analyses from GeparNuevo suggest that stromal TILs in residual disease may retain independent prognostic relevance, with patients exhibiting >10% residual stromal TILs showing substantially improved long-term outcomes compared with TIL-low counterparts (46). These findings support the concept that TILs function as a dynamic biomarker of ongoing immune surveillance across multiple time points, rather than as a fixed characteristic of the pre-treatment tumor (80).

Overall, TILs represent a biologically meaningful and guideline-endorsed prognostic biomarker in TNBC, yet their clinical utility remains constrained by methodological variability and the absence of standardized cut-offs. Their role as predictive biomarkers for immunotherapy selection remains insufficiently validated and cannot currently guide treatment decisions in routine practice (82). Resolving this gap will likely require integration with functional immune profiling and spatially resolved immune signatures to move beyond quantitative infiltration toward a more comprehensive characterization of immune competence within the tumor microenvironment. Furthermore, the practical implementation of standardized TIL assessment, while relatively low-cost compared with molecular assays, still requires trained pathologists and harmonized workflows that may not be universally available across healthcare settings, particularly in resource-limited environments.

### Spatial profiling and functional states

Emerging high-dimensional technologies, including spatial transcriptomics and single-cell RNA sequencing, may offer detailed insights into the localization, functional states, and intercellular interactions of immune cells within the TME (86–88). Beyond simple cellular density, the spatial organization of effector T cells, regulatory T cells, and myeloid populations relative to tumor nests and stromal compartments has been proposed to influence drug sensitivity (89).

Several landmark studies have begun to establish the potential clinical relevance of this spatial architecture. Danenberg et al. (90), employing imaging mass cytometry in 693 breast tumors, identified ten recurrent TME structures associated with distinct genomic profiles and clinical outcomes, including structures enriched for regulatory and dysfunctional T cells that appeared predictive of poor prognosis. Bassez et al. (91) applied single-cell RNA sequencing to paired pre- and on-treatment biopsies from breast cancer patients receiving neoadjuvant pembrolizumab, demonstrating that clonotypic expansion of CD8^+^ T cells with cytotoxic effector features during treatment was associated with pathological response. Beyond T cells, spatial profiling has begun to illuminate the potential contributions of tumor-associated macrophages (TAMs), dendritic cell niches, and myeloid-derived suppressor cells, which may either augment or suppress antitumor immunity depending on their localization and activation state (92, 93). B cells and tertiary lymphoid structures (TLS) have similarly been proposed as potentially important spatial and functional determinants of immunotherapy response, given their capacity to support antigen presentation, T-cell priming, and local immune activation within the TME (94). Functional immune states, spanning a spectrum from activated to exhausted or senescent phenotypes, may be further modulated by tumor-intrinsic signaling pathways, including WNT/β-catenin and PI3K/AKT, as well as by metabolic constraints and the local cytokine milieu (95).

Despite these advances, the clinical implementation of spatial transcriptomics remains hampered by substantial technical and logistical barriers. Most platforms have historically required fresh-frozen tissue, posing significant challenges for integration into routine formalin-fixed paraffin-embedded diagnostic workflows, although recent methodological developments may begin to address some of these limitations (96, 97). Furthermore, the absence of standardized bioinformatic pipelines, combined with high costs and prolonged turnaround times, currently limits applicability in time-sensitive clinical contexts such as neoadjuvant treatment decision-making (98). At present, therefore, spatial profiling technologies appear best positioned as research tools for biomarker discovery within prospective clinical trials and well-annotated translational cohorts, rather than for routine clinical deployment.

## Emerging molecular biomarkers

Beyond immune-related biomarkers, several molecular markers have emerged as potential tools for improving risk stratification and therapeutic decision-making in TNBC (**Table 3**). These include more advanced clinically validated approaches, such as circulating tumor DNA (ctDNA), as well as investigational biomarkers, including oncogenic pathway signatures and microRNAs (miRNAs), which may further characterize tumor biology and disease evolution (**Figure 3**).

### Gene expression–based biomarkers

Gene expression–based signatures have emerged as a complementary approach to conventional biomarkers, with the aim of capturing the functional complexity of the tumor microenvironment beyond static histopathological assessment. In triple-negative breast cancer (TNBC), transcriptomic analyses have enabled the development of molecular stratification strategies that identify distinct tumor subgroups characterized by different immune and proliferative profiles. However, despite these advances in tumor classification, their translation into clinical practice remains limited (21–26).

Beyond these subgrouping efforts, TNBC-DX, a recently developed multigene assay, has gained attention as a promising tool for early-stage TNBC. TNBC-DX integrates immune- and proliferation-related gene expression modules with key clinicopathological variables, including tumor size and nodal status, to generate two complementary outputs. The first is a pathological complete response (pCR) score, which estimates the likelihood of response to neoadjuvant therapy. The second is a risk score, which predicts long-term clinical outcomes. In the study by Martín et al. (99), retrospective validation cohorts of patients treated with neoadjuvant taxane-based chemotherapy without pembrolizumab demonstrated increasing pCR rates across pCR-low, pCR-medium, and pCR-high categories (22.5%, 53.6%, and 56.3%, respectively). Moreover, the risk score was independently associated with DDFS and OS. In an independent cohort of patients receiving pembrolizumab-based neoadjuvant chemoimmunotherapy, including patients enrolled in the NeoPACT trial, both scores retained significant associations with pCR, EFS and OS (99).

Although current evidence is derived from retrospective analyses of samples collected within previously conducted clinical trials and institutional cohorts, TNBC-DX has consistently demonstrated both prognostic and predictive value across multiple independent datasets. Among currently available gene expression–based assays for early-stage TNBC, TNBC-DX is the most mature and clinically translatable tool, with the strongest evidence supporting its potential short- to mid-term implementation in clinical practice. Nevertheless, prospective interventional studies demonstrating its clinical utility for treatment selection and improved patient outcomes are still lacking. Further prospective validation will be essential to define its role in routine clinical practice and future guideline recommendations.

### Oncogenic signaling pathways

Aberrant activation of tumor-intrinsic oncogenic signaling pathways has been proposed to contribute substantially to immune evasion in TNBC. The PI3K/AKT/mTOR pathway, which is frequently altered through PIK3CA mutations, PTEN loss, or AKT amplification, has been implicated in promoting tumor proliferation and survival while potentially shaping an immunosuppressive tumor microenvironment (100–103). Similarly, activation of MAPK signaling has been associated with reduced chemokine-mediated T-cell recruitment, while WNT/β-catenin signaling has been associated with immune-cold phenotypes characterized by reduced dendritic cell recruitment and lower intratumoral immune infiltration (101–104). Although these observations provide a biologically plausible framework linking oncogenic signaling to antitumor immunity, most evidence derives from preclinical models and retrospective translational studies, with limited prospective validation in early-stage TNBC.

In early TNBC treated with neoadjuvant therapy, oncogenic pathway activation signatures have primarily been investigated as potential biomarkers of resistance to chemotherapy and immune checkpoint blockade. Exploratory biomarker analyses from KEYNOTE-522 suggested that tumors characterized by immune-suppressed or poorly inflamed transcriptional programs were associated with lower rates of pCR and reduced benefit from neoadjuvant chemo-immunotherapy (11, 18). Additional transcriptomic analyses have reported associations between PI3K/AKT, MAPK, and β-catenin signaling signatures and reduced TIL density, lower interferon-γ–related gene expression, and inferior pathological response rates (38), providing translational support for the immune-inactive tumor phenotypes suggested by preclinical models. However, these findings remain largely correlative and have not been prospectively validated using standardized pathway-specific biomarkers. Consequently, no oncogenic signaling signature is currently recommended for treatment selection or risk stratification in routine clinical practice.

Clinical efforts to therapeutically target oncogenic pathways in TNBC through inhibition of the PI3K/AKT axis have been explored across both metastatic and early-stage disease, although evidence remains limited (**Supplementary Table 1**). Most clinical experience derives from the metastatic setting, where early studies suggested potential activity of AKT inhibitors, however, results across subsequent randomized trials have been heterogeneous, and no consistent benefit has been demonstrated across biomarker-selected populations (105–108). These findings highlight the biological complexity of TNBC and the limitations of current pathway-based patient selection strategies. Evidence in early-stage TNBC remains substantially more limited. In the phase II FAIRLANE trial, the addition of ipatasertib to paclitaxel increased radiological response rates but did not significantly improve pCR compared with paclitaxel alone (pCR 17% versus 13% in the ITT population) (109). To date, no completed phase II or III neoadjuvant or post-neoadjuvant study has demonstrated a clear clinical benefit for PI3K/AKT pathway inhibition in early TNBC.

Given the limited clinical benefit observed with PI3K/AKT pathway inhibition, interest has expanded toward alternative oncogenic networks that may contribute to treatment resistance. Strategies targeting MAPK signaling, including MEK inhibition, have been investigated based on preclinical evidence suggesting a role in tumor progression and resistance to systemic therapy (110). Similarly, AR-associated networks have attracted interest, particularly in the LAR subtype of TNBC, where preclinical studies have suggested functional crosstalk between AR and PI3K/AKT signaling pathways that may contribute to adaptive resistance mechanisms. However, clinical studies evaluating these approaches, either alone or in combination with chemotherapy, immunotherapy, or PI3K pathway inhibitors, have generally reported modest and inconsistent activity, with limited evidence of durable benefit (111, 112).

### Circulating tumor DNA as a marker of minimal residual disease

ctDNA has emerged as one of the most clinically advanced dynamic biomarkers in TNBC, with its principal potential advantage lying in enabling real-time, non-invasive longitudinal monitoring throughout the clinical course (**Table 2**). Two major analytical strategies are currently employed. Tumor-informed assays, based on patient-specific somatic variants identified in the primary tumor, may enable highly sensitive MRD detection even at variant allele frequencies below the threshold of conventional imaging or pathology (55, 56). Tumor-agnostic assays, by contrast, rely on predefined gene panels and do not require prior tumor sequencing; although more scalable, they generally exhibit lower analytical sensitivity and specificity for MRD detection (113, 114). Heterogeneity in assay performance and the current absence of cross-platform standardization represent important limitations common to both approaches (115).

In multiple studies, ctDNA has demonstrated incremental prognostic value, particularly when integrated with established pathological parameters. In a prospective multisite registry of TNBC patients with residual disease following neoadjuvant therapy, both RCB class and ctDNA status were independently associated with outcomes (RCB: HR 5.16; ctDNA: HR 3.71), with ctDNA appearing to further refine prognostic discrimination within RCB-II disease (3-year EFS: 65% vs 87%) (116). The prognostic relevance of ctDNA is further influenced by the timing of assessment. Baseline levels may provide independent prognostic information, on-treatment dynamics may reflect early therapeutic response or emerging resistance, and postoperative ctDNA status may refine risk stratification beyond conventional pathological parameters, although the latter hypothesis awaits prospective confirmation (117).

Multiple studies have suggested that ctDNA positivity following curative-intent treatment may precede clinical or radiological relapse by several months, potentially offering a therapeutic window for early intervention (55, 56, 118, 119). In a prospective study of early breast cancer, post-treatment ctDNA detection was associated with metastatic recurrence with a median lead time of approximately 7–10 months before clinical relapse became evident (56). In the BRE12–158 randomized clinical trial, combined assessment of ctDNA and circulating tumor cells after neoadjuvant chemotherapy was associated with risk stratification of disease recurrence in TNBC, supporting the hypothesis that liquid biopsy–based markers may capture residual systemic disease not fully reflected by imaging or pathology (120). Notably, persistent or re-emergent ctDNA after surgery has been associated with an increased risk of relapse even among patients achieving pCR by conventional pathology, highlighting the potential complementary value of molecular residual disease assessment over pathological response alone (56, 119).

Beyond its prognostic role, ctDNA is increasingly being investigated as a biomarker to inform therapeutic decision-making. In the post-neoadjuvant setting, ctDNA has been incorporated into clinical trial designs as a tool for risk stratification, patient enrichment, or MRD assessment (**Supplementary Table 2**). Ongoing studies are evaluating MRD-guided strategies, including PERSEVERE (NCT04849364), the phase III randomized ZEST trial (NCT04915755), and the NSABP B-59/GBG-96-GeparDouze MRD substudy (NCT03281954). However, whether ctDNA-guided treatment adaptation can improve clinical outcomes remains uncertain, as prospective interventional evidence demonstrating clinical benefit is still limited.

Despite this growing clinical interest, implementation of ctDNA testing in routine practice remains limited by important methodological and biological constraints, including inter-assay variability, reduced sensitivity in low-shedding tumors, and potential false-positive results driven by clonal hematopoiesis of indeterminate potential (CHIP). The absence of standardized analytical frameworks and validated positivity thresholds further limits its readiness for routine clinical deployment. Beyond analytical and biological constraints, significant disparities exist in access to ctDNA testing globally. High costs, limited reimbursement frameworks, and the absence of validated assays in low- and middle-income countries represent major barriers to equitable implementation. Until cost-effective, standardized platforms become widely available, the clinical utility of ctDNA-based strategies will remain largely confined to well-resourced academic settings, potentially widening existing inequalities in cancer care.

### MicroRNAs as biomarkers of prognosis and treatment response in TNBC

miRNAs are small non-coding RNAs that regulate gene expression through post-transcriptional mechanisms and have emerged as important modulators of cancer biology. In TNBC, miRNAs have been implicated in multiple processes relevant to tumor progression, including proliferation, EMT, apoptosis, angiogenesis, immune regulation, and DNA damage response pathways (121–124). Their ability to simultaneously regulate multiple targets across interconnected signaling networks has generated interest in their potential role as biomarkers of prognosis and treatment response.

Several miRNAs have been implicated in the regulation of tumor cell-intrinsic pathways and immune microenvironment components in TNBC (125–130). Tumor-suppressive miRNAs, including miR-34a (125, 126) and members of the miR-200 family (128), have been associated with regulation of EMT-related programs and PD-L1 expression through preclinical models, while oncogenic miRNAs such as miR-21 and miR-155 have been linked to pro-tumorigenic inflammatory signaling (129, 130). However, the clinical relevance of these findings in TNBC remains to be established.

The prognostic significance of miRNA dysregulation in breast cancer has been evaluated in multiple retrospective studies and meta-analyses (**Supplementary Table 3**). Among the various candidates investigated, miR-21 overexpression has shown the most consistent association with adverse clinical outcomes, while miR-27a/b and miR-155 have demonstrated less consistent results across studies (131, 132). Conversely, higher expression of miR-374a/b has been associated with more favorable outcomes in exploratory analyses. More recently, global miRNA profiling approaches have identified novel candidates, including miR-3916 and miR-3613-5p, which may improve prediction of metastatic recurrence when integrated with clinicopathological variables (133).

Interest has also expanded toward circulating miRNAs as minimally invasive biomarkers. Their remarkable stability in plasma and serum, largely attributable to encapsulation within extracellular vesicles or association with RNA-binding proteins, makes them attractive candidates for liquid biopsy applications (134, 135). Exploratory studies have suggested associations between circulating miRNA signatures and recurrence risk, treatment response, and pathological complete response following neoadjuvant therapy (136–138) (**Supplementary Table 3**). These findings raise the possibility that circulating miRNAs could complement other dynamic biomarkers, such as ctDNA, in monitoring residual disease and treatment efficacy.

Despite substantial biological interest, miRNAs remain investigational biomarkers. Clinical translation is currently limited by heterogeneity in study design, analytical methodologies, normalization strategies, and patient populations, as well as the predominance of retrospective evidence (139). Consequently, prospective validation and methodological standardization will be essential before miRNA-based biomarkers can be incorporated into routine clinical decision-making in TNBC.

## Conclusions and future perspectives

The therapeutic landscape of early-stage TNBC has been fundamentally reshaped by the incorporation of ICIs into the neoadjuvant setting. Despite these advances, a substantial proportion of patients continue to experience residual disease and disease recurrence, underscoring the persistence of major unmet clinical needs. Current clinical decision-making remains largely anchored to static post-treatment endpoints such as pCR and RCB, and complemented by conventional clinicopathological variables (**Figure 2**). However, this framework does not fully capture the pronounced molecular heterogeneity and dynamic tumor–immune interactions that characterize TNBC. A major unmet need remains the absence of validated biomarkers capable of guiding real-time treatment adaptation across the disease continuum. Addressing this gap will require a shift toward integrated, longitudinal biomarker strategies particularly those incorporating tissue-based and liquid biopsy-derived metrics of MRD (**Figure 3**). Successful clinical implementation will depend on rigorous analytical validation, cross-platform standardization, and harmonized definitions of MRD, as well as prospective evidence demonstrating that biomarker-guided treatment adaptation improves outcomes without increasing overtreatment or toxicity burden.

Among clinically actionable near-term priorities, ctDNA-based MRD assessment represents the most advanced emerging strategy, with several prospective interventional trials currently evaluating whether MRD-guided treatment escalation or de-escalation can improve outcomes in the post-neoadjuvant setting. The integration of RCB with molecular residual disease metrics may further refine post-surgical risk stratification and inform adjuvant treatment decisions, although prospective validation of combined models is still required. Standardization of TILs assessment and its integration into routine pathological workflows also represents a clinically feasible near-term objective, given its established prognostic value and relatively low implementation cost compared to molecular assays.

A major future challenge in TNBC precision oncology is the translation of emerging biomarkers into clinically actionable strategies through the integration of MRD assessment, multi-omics profiling, and adaptive clinical trial designs. Artificial intelligence (AI)-driven models integrating digital pathology, genomic data, immune signatures, and liquid biopsy dynamics may improve risk stratification and refine prediction of treatment response; however, prospective validation in well-annotated, representative cohorts remains a prerequisite before clinical deployment. In parallel, multi-omics approaches may enhance biological resolution by characterizing convergent oncogenic dependencies across genomic, transcriptomic, proteomic, and epigenomic layers, although challenges related to data integration, interpretability, and standardization remain substantial. These methodological advances are best regarded as medium- to long-term research priorities rather than near-term clinical tools. Among emerging computational paradigms, federated learning may help overcome barriers to data sharing by enabling decentralized model development while preserving patient privacy and regulatory compliance. Similarly, biomarker-driven adaptive platform trials represent a methodologically important advance to accelerate the clinical validation of predictive biomarkers and treatment strategies in a more efficient and flexible framework. Integration of real-world data, including registries, electronic health records, and patient-reported outcomes, will also be essential to ensure external validity and to better capture the clinical heterogeneity that characterizes patients treated outside controlled trial settings.

Despite these advances, significant translational and structural barriers remain. The implementation of multi-omics technologies, AI infrastructure, and longitudinal biomarker monitoring is resource-intensive and may potentially exacerbate disparities in cancer care, particularly in low- and middle-income countries. Reimbursement limitations, lack of infrastructure for biospecimen processing, and restricted access to next-generation sequencing platforms further constrain the applicability of precision oncology frameworks in these settings. Addressing these disparities will require coordinated efforts involving regulatory bodies, payers, and the global oncology community to develop context-adapted, resource-stratified biomarker strategies. Particular attention should be directed toward the development of cost-effective, analytically validated assays that can be implemented across diverse healthcare settings, ensuring that advances in precision oncology translate into equitable improvements in patient outcomes. Moreover, limited access to high-quality molecular diagnostics and potential biases in data representation may further constrain the generalizability of precision oncology approaches across diverse populations.

In conclusion, the major unmet need in early-stage TNBC remains the development of clinically validated, dynamic biomarker frameworks capable of enabling response-adaptive treatment strategies and more precise therapeutic personalization across the disease continuum. Achieving this goal will require not only technological innovation, but also robust clinical validation, methodological standardization, and equitable implementation strategies to ensure broad, sustainable, and clinically meaningful impact.
